# From Tissue Signaling to Liquid Biopsy: A pAKT-Driven microRNA Model for Oral Squamous Cell Carcinoma Detection

**DOI:** 10.7759/cureus.115008

**Published:** 2026-08-22

**Authors:** Sanjana Gupta, Sajid Pervez

**Affiliations:** 1 Department of Oral and Maxillofacial Pathology and Microbiology, I.T.S. Centre for Dental Studies and Research, Ghaziabad, IND; 2 Family Medicine and Primary Care, Wellness Clinic, New Delhi, IND

**Keywords:** microrna-126, microrna-196a1, oral carcinogenesis, oral potentially malignant disorders, oral squamous cell carcinoma, phosphorylated akt (pakt), pi3k/akt signaling

## Abstract

Background

Oral squamous cell carcinoma (OSCC) develops through a multistep progression from oral potentially malignant disorders (OPMDs), yet reliable biomarkers that reflect the molecular mechanisms driving malignant transformation are lacking. Aberrant activation of the PI3K/AKT pathway is a key event in epithelial carcinogenesis, but its integration with upstream regulatory microRNAs (miRNAs) in oral cancer progression remains poorly understood.

Methodology

Phosphorylated AKT (pAKT) expression was evaluated by immunohistochemistry in oral epithelial tissues spanning the OPMD-OSCC continuum. In parallel, circulating serum levels of the oncogenic microRNA miR-196a1 and the tumor-suppressive microRNA miR-126 were quantified using quantitative real-time PCR. Associations between tissue pAKT expression, circulating miRNA levels, and clinicopathological parameters were assessed. Diagnostic performance of individual and combined circulating miRNA markers for distinguishing OSCC from OPMDs was evaluated using receiver operating characteristic analysis and multivariable logistic regression

Results

Tissue analysis revealed a progressive increase in pAKT expression with advancing histological severity, indicating a stepwise activation of the PI3K/AKT signaling pathway during malignant transformation. Serum profiling revealed significant upregulation of miR-196a1 and concomitant downregulation of miR-126 across disease progression, with strong associations to histological grade and tobacco exposure. Loss of miR-126, a negative regulator of PI3K signaling, showed marked biological concordance with increased tissue pAKT expression. Integration of tissue pAKT status with reciprocal microRNA dysregulation enabled robust discrimination between premalignant and malignant lesions with strong internal validation.

Conclusion

This study identifies a novel pAKT-microRNA regulatory axis in oral carcinogenesis, wherein oncogenic gain of miR-196a1 and loss of miR-126 converge on PI3K/AKT pathway activation. Integration of tissue pAKT alterations with circulating microRNA profiles provides mechanistic context for the observed molecular differences across OPMDs and OSCC. The combined circulating miRNA model demonstrated strong discriminatory performance for distinguishing OSCC from OPMDs, supporting further validation of this tissue-circulating biomarker framework in independent cohorts.

## Introduction

Oral squamous cell carcinoma (OSCC) is a common malignancy affecting the oral cavity and is associated with significant morbidity and mortality worldwide. OSCC accounts for a substantial number of new cancer cases annually and presents considerable challenges in clinical management because of late diagnosis and the unpredictable progression of oral potentially malignant disorders (OPMDs), including leukoplakia and oral lichen planus. Early detection and risk stratification remain limited because of the lack of reliable molecular biomarkers that accurately reflect disease progression [1].

Dysregulation of the phosphoinositide 3-kinase/protein kinase B (PI3K/AKT) signaling pathway plays an important role in OSCC pathogenesis. Activation of this pathway promotes cellular proliferation, survival, and resistance to apoptosis. Phosphorylated AKT (pAKT) is a key indicator of PI3K/AKT pathway activation and has been associated with tumor progression and therapeutic resistance in OSCC [2]. However, previous studies have primarily evaluated pathway alterations independently without integrating upstream regulatory mechanisms or circulating biomarkers across the disease spectrum.

MicroRNAs (miRNAs) are endogenous non-coding RNAs that regulate gene expression at the post-transcriptional level and participate in multiple oncogenic and tumor-suppressive pathways. Altered miRNA expression has been widely reported in OSCC and has been associated with tumor progression, invasion, and metastasis [3]. Circulating miRNAs are detectable in serum and plasma and exhibit high biological stability, making them promising candidates for non-invasive biomarker development. However, many studies have focused on individual miRNAs without evaluating their relationship with specific signaling pathways.

Among the miRNAs implicated in OSCC, miR-196a1 has been associated with increased cellular proliferation, invasion, and malignant progression [4]. In contrast, miR-126 has been reported to regulate components of the PI3K/AKT pathway, and reduced expression has been associated with enhanced tumor progression in epithelial malignancies [5]. Despite these findings, the relationship between miR-196a1, miR-126, and pAKT expression during OSCC progression has not been comprehensively evaluated.

A major challenge in oral oncology is the identification of biomarkers that correlate molecular alterations in tissue with non-invasive circulating biomarkers. Tissue immunohistochemistry provides information regarding signaling pathway activation but requires invasive sampling. Conversely, circulating biomarkers are minimally invasive but may lack direct correlation with tissue-level molecular alterations. Therefore, the present study aimed to evaluate tissue pAKT expression and circulating levels of miR-196a1 and miR-126 across the spectrum of OPMDs and OSCC, assess their associations with clinicopathological characteristics and with each other, and evaluate the diagnostic performance of individual and combined circulating miRNA markers for distinguishing OSCC from OPMDs. The study further sought to interpret the relationship between tissue pAKT activation and circulating miRNA dysregulation within a biologically integrated framework.

## Materials and methods

Study design and ethical approval

This analytical cross-sectional study was conducted in the Department of Oral and Maxillofacial Pathology and Microbiology at I.T.S. Centre for Dental Studies and Research, situated in Murad Nagar, Uttar Pradesh, India. The study received approval from the Institutional Ethics Committee of I.T.S. Centre for Dental Studies and Research (Approval/Protocol No.: ITSCDSR/IEC/RP/2021/005), and the necessary infrastructure support was provided by the Indian Council of Medical Research (ICMR) - National Institute of Cancer Prevention and Research. Written informed consent was obtained from all participants prior to their involvement in the study. The research followed the ethical principles outlined in the Declaration of Helsinki.

Study population and sample selection

A total of 150 subjects were enrolled and categorized into oral potentially malignant disorders (OPMDs; n = 100) and oral squamous cell carcinoma (OSCC; n = 50). OPMDs included oral leukoplakia (n = 50), oral lichen planus (n = 19), and oral submucous fibrosis (n = 31). OSCC cases were histopathologically graded as well-differentiated (n = 24), moderately differentiated (n = 12), and poorly differentiated (n = 14). Cases with inadequate tissue, incomplete clinical data, or a history of chemotherapy or radiotherapy were excluded. Demographic details and habit history, including tobacco type, duration, and frequency, were systematically recorded.

Sample size justification

The sample size for this study was determined based on anticipated differences in biomarker expression between OPMDs and OSCC, informed by previously published studies on oral cancer biomarkers evaluating tissue signaling markers and circulating miRNAs. Prior studies have reported large effect sizes in the differential expression of oncogenic and tumor-suppressive miRNAs across malignant transformation, justifying a moderate sample size for adequate statistical power. Assuming a large effect size (Cohen’s d ≥ 0.80), a two-sided significance level (α) of 0.05, and a statistical power of 80%, a minimum of approximately 40-45 subjects per group was estimated to be sufficient to detect biologically meaningful differences in biomarker expression between OPMD and OSCC groups. To enhance statistical robustness, allow subgroup analyses across different OPMD categories and histological grades, and reduce the risk of model instability in multivariable analyses, a larger cohort of 150 participants (OPMDs = 100; OSCC = 50) was recruited. This sample size was considered adequate for exploratory integrative biomarker modeling, including logistic regression and receiver operating characteristic (ROC) analyses, while maintaining acceptable events-per-variable ratios for internal model validation.

Immunohistochemical evaluation of pAKT

Formalin-fixed paraffin-embedded (FFPE) tissue sections (4 µm) were mounted on poly-L-lysine-coated slides, deparaffinized, and rehydrated through graded alcohols. Antigen retrieval was performed using citrate buffer (pH 6.0) in a pressure cooker for 20 minutes. Endogenous peroxidase activity was blocked using 3% hydrogen peroxide, followed by incubation with normal serum to reduce nonspecific binding. Sections were incubated overnight at 4 °C with a monoclonal antibody against phosphorylated AKT (pAKT, Ser473; Cell Signaling Technology, Danvers, MA, USA; Cat. No. 4060), according to the manufacturer's recommended conditions. Detection was performed using the streptavidin-biotin peroxidase technique with diaminobenzidine (DAB) as chromogen, and slides were counterstained with haematoxylin. Normal oral epithelium served as an internal positive control, while negative controls were prepared by omitting the primary antibody.

Assessment of pAKT expression

pAKT immunoreactivity was independently evaluated by two blinded oral pathologists in five randomly selected high-power fields (400×) per section. Cytoplasmic and nuclear staining patterns were recorded. Quantitative assessment involved estimating the proportion of pAKT-positive cells, while qualitative evaluation was based on staining intensity scored as 0 (negative), 1 (weak), 2 (moderate), or 3 (strong). Inter-observer agreement was assessed using Cohen’s kappa statistic, and discrepant scores were resolved by consensus.

Serum collection and RNA isolation

Peripheral venous blood (5 mL) was collected under aseptic conditions, allowed to clot at room temperature, and centrifuged at 2000 rpm for 10 minutes. Serum was carefully separated, aliquoted into RNase-free tubes, and stored at −80 °C. Total RNA, including small RNA fractions, was extracted from 200 µL of serum using TRI Reagent LS (Sigma-Aldrich, St. Louis, MO, USA; Cat. No. T3934) following the manufacturer’s protocol. RNA concentration and purity were assessed using NanoDrop (Thermo Fisher Scientific, Waltham, MA, USA) spectrophotometry, and samples with A260/A280 ratios between 1.8 and 2.0 were considered acceptable for downstream analysis.

cDNA synthesis and quantitative real-time PCR

Complementary DNA (cDNA) was synthesized from 200 ng of total RNA using the High-Capacity cDNA Reverse Transcription Kit (Applied Biosystems, Thermo Fisher Scientific). Quantitative real-time PCR was performed using SYBR Green chemistry on an Applied Biosystems QuantStudio™ 3 Real-Time PCR system. Specific primers for miR-196a1, miR-126, and endogenous control U6 snRNA were designed using miRBase and validated through Primer-BLAST. Thermal cycling conditions included an initial denaturation at 95 °C for 10 minutes, followed by 40 cycles of 95 °C for 15 seconds and 60 °C for 60 seconds. All reactions were performed in triplicate. Melt-curve analysis confirmed amplification specificity. Relative miRNA expression was calculated using the 2⁻ΔΔCt method, normalized to U6 snRNA, with healthy controls used as the calibrator.

Statistical analysis

Statistical analyses were performed using IBM SPSS version 23.0 (IBM Corp., Armonk, NY, USA), GraphPad Prism version 9.0 (La Jolla, CA, USA), and R version 4.2.1 (R Foundation for Statistical Computing, Vienna, Austria). Data distribution was assessed using the Shapiro-Wilk test, and homogeneity of variance was evaluated using Levene’s test. Group comparisons were performed using one-way analysis of variance (ANOVA) followed by Tukey’s post-hoc test or appropriate non-parametric tests where applicable. Correlations between tissue pAKT expression, circulating miR-196a1 and miR-126 levels, and clinicopathological parameters were assessed using Spearman’s rank correlation analysis. Diagnostic performance was evaluated using ROC analysis and multivariable logistic regression. A p-value < 0.05 was considered statistically significant.

## Results

A total of 150 subjects were included, comprising 100 cases of OPMDs and 50 cases of OSCC. Patients with OSCC were relatively older (60% aged ≥50 years vs. 50% in OPMDs), but the difference was not statistically significant (p = 0.298). A male predominance was observed in both groups (OPMDs: 92%; OSCC: 98%; p = 0.273). Tobacco habit type differed significantly between groups (p < 0.001). Combined smoking and smokeless tobacco use was more frequent in OSCC (54%) compared to OPMDs (27%). Duration of tobacco exposure was significantly higher in OSCC patients (≥50 years: 48% vs. 1%; p < 0.001). Frequency of tobacco use was also higher in OSCC (≥1/day: 94% vs. 35%; p < 0.001) (Table 1).

**Table 1:** Clinicodemographic and Habit Characteristics of Study Participants A comparison of clinical and demographic features, as well as tobacco usage patterns, was conducted between oral potentially malignant disorders (OPMDs) and oral squamous cell carcinoma (OSCC). The data is displayed as numbers (percentages). The chi-square test (or Fisher's exact test when appropriate) was used to evaluate statistical significance. A p-value of less than 0.05 was regarded as statistically significant.

Variable	OPMDs (n=100)	OSCC (n=50)	χ² value	p-value
Age (years)			1.09	0.298
<50	50 (50%)	20 (40%)		
≥50	50 (50%)	30 (60%)		
Sex			1.21	0.273
Male	92 (92%)	49 (98%)		
Female	8 (8%)	1 (2%)		
Tobacco habit type			18.64	<0.001
Smoking only	56 (56%)	23 (46%)		
Smokeless only	17 (17%)	0 (0%)		
Smoking + smokeless	27 (27%)	27 (54%)		
Duration of tobacco exposure			46.88	<0.001
<50 years	99 (99%)	26 (52%)		
≥50 years	1 (1%)	24 (48%)		
Frequency of tobacco use			50.67	<0.001
<1/day	65 (65%)	3 (6%)		
≥1/day	35 (35%)	47 (94%)		

Histopathological distribution

**Figure 1:**
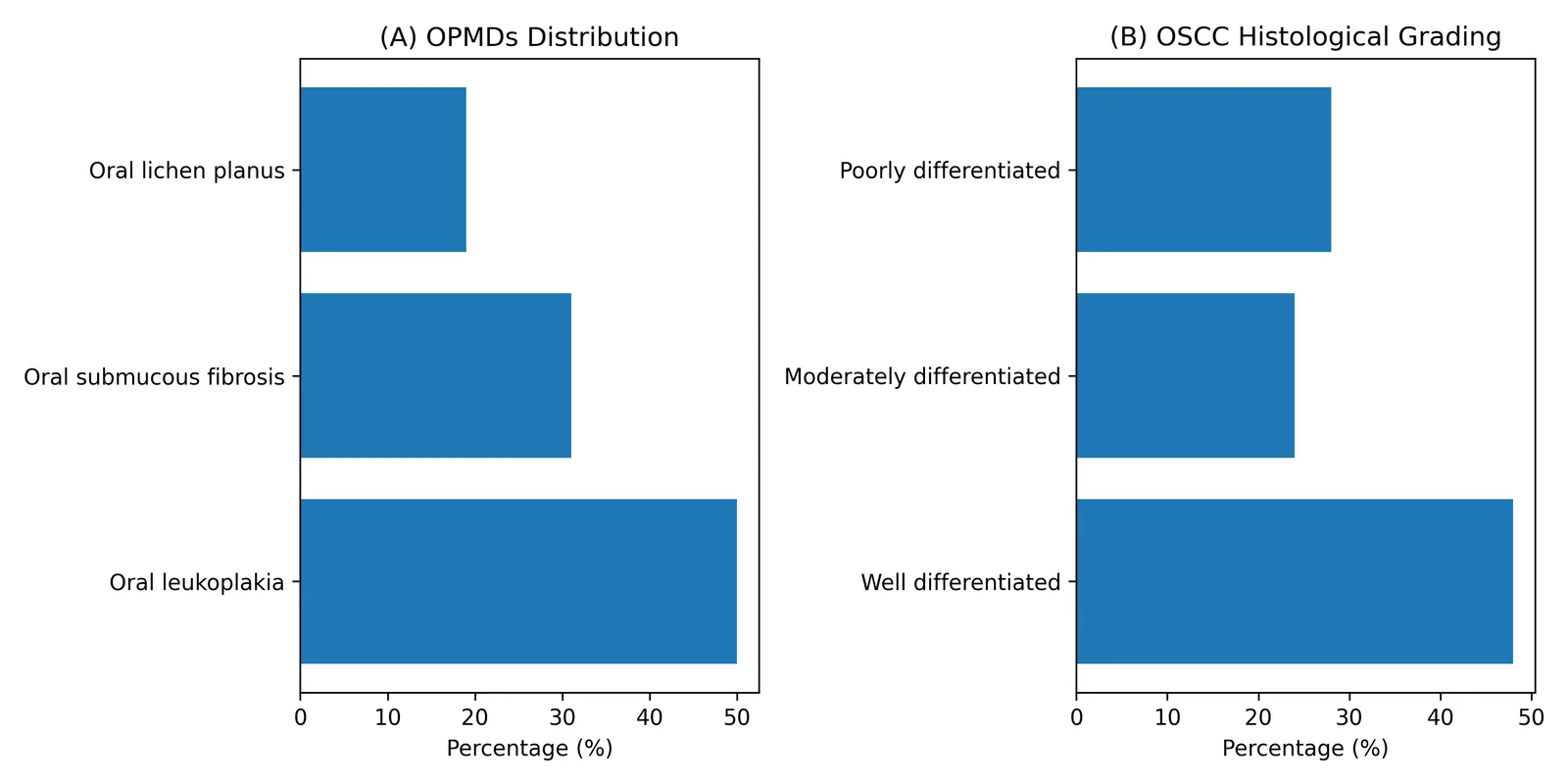
Diagnostic Distribution of Oral Potentially Malignant Disorders and Histological Grading of Oral Squamous Cell Carcinoma Diagnostic classification of oral potentially malignant disorders and histological grading of oral squamous cell carcinoma. (A) Distribution of oral potentially malignant conditions (OPMCs), such as oral leukoplakia, oral submucous fibrosis, and oral lichen planus. (B) Histological classification of oral squamous cell carcinoma (OSCC), encompassing well-differentiated, moderately differentiated, and poorly differentiated carcinoma.

Among OPMDs, oral leukoplakia was the most common lesion (50%), followed by oral submucous fibrosis (31%) and oral lichen planus (19%). OSCC cases were categorized as well-differentiated (48%), moderately differentiated (24%), and poorly differentiated (28%). The distribution differed significantly between groups (p < 0.001) (Figure 1).

pAKT expression in OPMDs and OSCC

Quantitative Analysis

OPMDs showed low pAKT expression (mean ± SD: 0.03 ± 0.13; median [IQR]: 0). OSCC showed higher pAKT expression (mean ± SD: 12.0 ± 3.72; median [IQR]: 11). The difference between groups was statistically significant (p < 0.001) (Table 2).

**Table 2:** Quantitative Immunohistochemical Evaluation of pAKT Expression Quantitative comparison of pAKT-positive cell expression between oral potentially malignant disorders (OPMDs) and oral squamous cell carcinoma (OSCC). Data are presented as mean ± standard deviation (SD) and median with interquartile range (IQR). Statistical significance was assessed using the Mann-Whitney U test. A p-value < 0.05 was considered statistically significant.

Diagnostic Category	Mean pAKT-Positive Cells ± SD	Median	IQR	Test Statistic (Z value)	p-value
OPMDs	0.03 ± 0.13	0	0	-10.42	<0.001
OSCC	12.0 ± 3.72	11	3		

Qualitative Analysis

**Figure 2:**
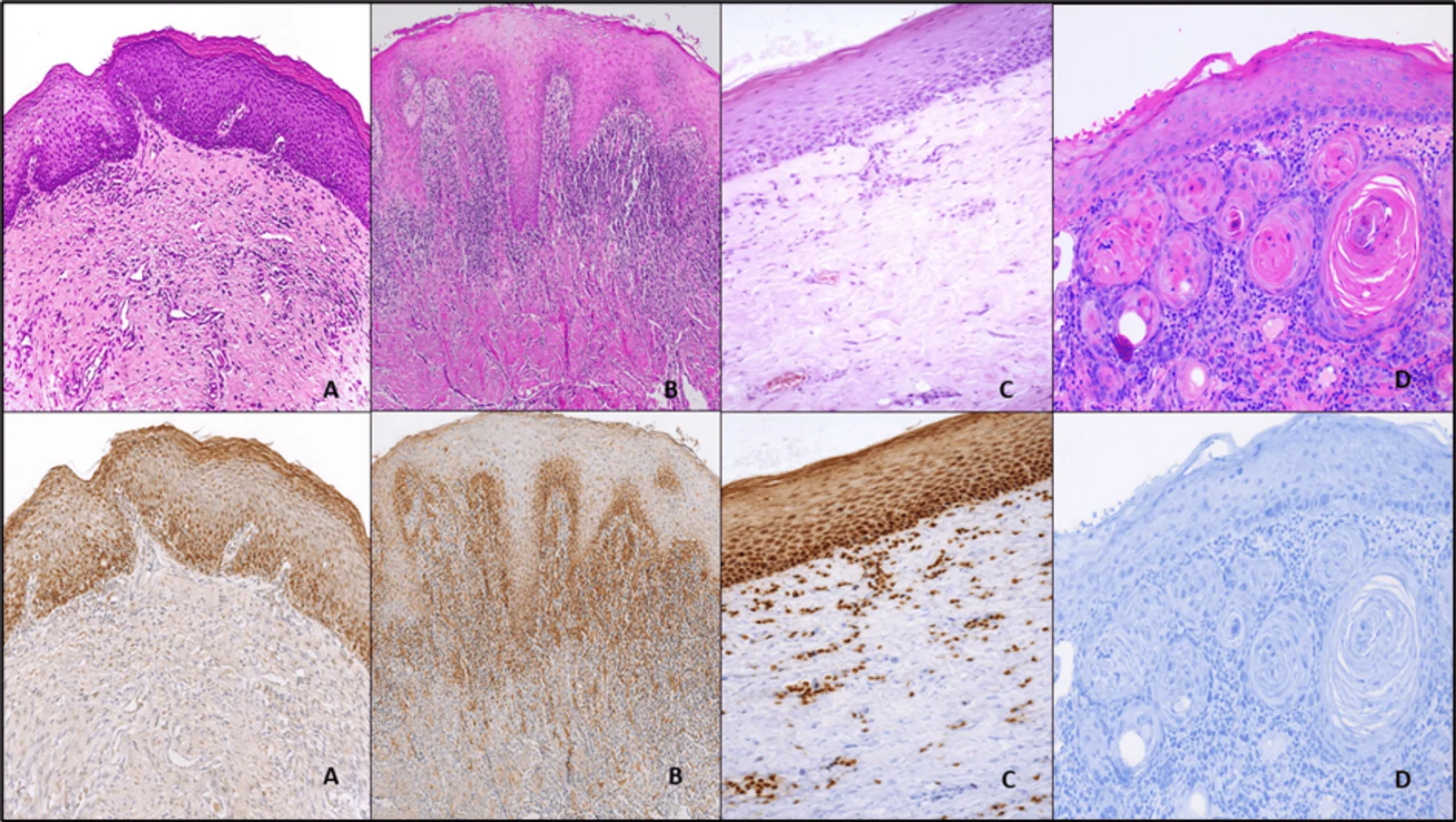
Histopathological and Immunohistochemical Analysis of Oral Potentially Malignant Disorders and Oral Squamous Cell Carcinoma. (A-D) H&E-stained sections illustrate: (A) oral leukoplakia with hyperkeratosis and epithelial thickening, (B) oral lichen planus with basal cell degeneration and subepithelial inflammation, (C) oral submucous fibrosis characterized by epithelial atrophy and dense collagen deposition, and (D) oral squamous cell carcinoma featuring invasive malignant squamous cell nests with keratin pearl formation. Immunohistochemical staining for pAKT is also included.

Most OPMDs showed weak or absent staining. In OSCC, 92% of cases showed strong staining, and 8% showed mild staining. No cases showed moderate or negative staining. The difference between groups was statistically significant (p < 0.001) (Figure 2).

Risk estimation of pAKT overexpression

**Figure 3:**
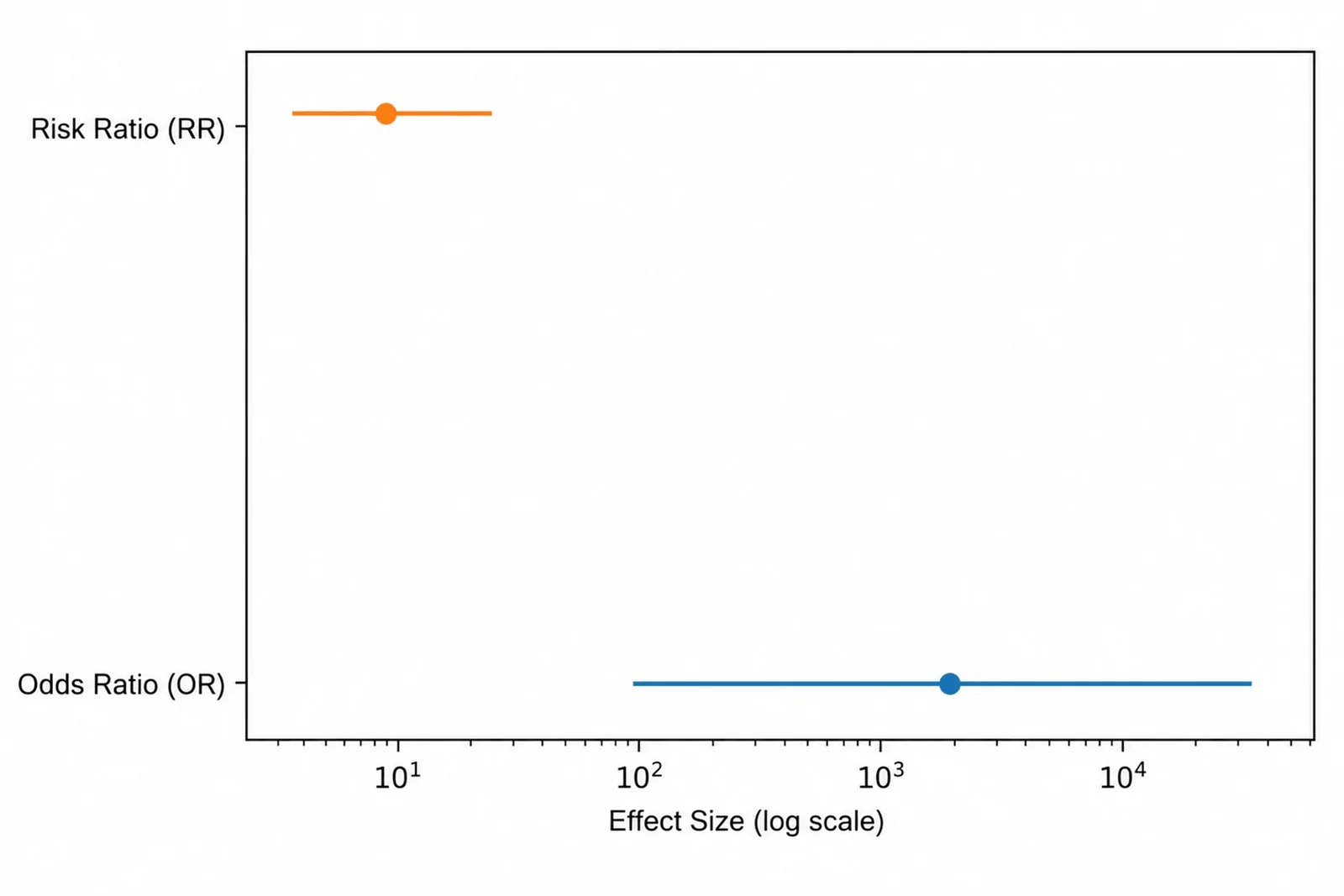
Evaluation of the Risk Associated With pAKT Overexpression in Oral Squamous Cell Carcinoma in Relation to Oral Potentially Malignant Disorders. The forest plot displays the odds ratio (OR) and risk ratio (RR) for the overexpression of pAKT in oral squamous cell carcinoma (OSCC) in comparison to oral potentially malignant disorders (OPMDs). Effect sizes are shown on a logarithmic scale along with their corresponding 95% confidence intervals.

The odds ratio for strong pAKT expression in OSCC compared to OPMDs was 2077.0 (95% CI: 109.5-39,380.6). The relative risk was 11.28 (95% CI: 4.67-27.25) (Figure 3).

Diagnostic accuracy of pAKT

**Figure 4:**
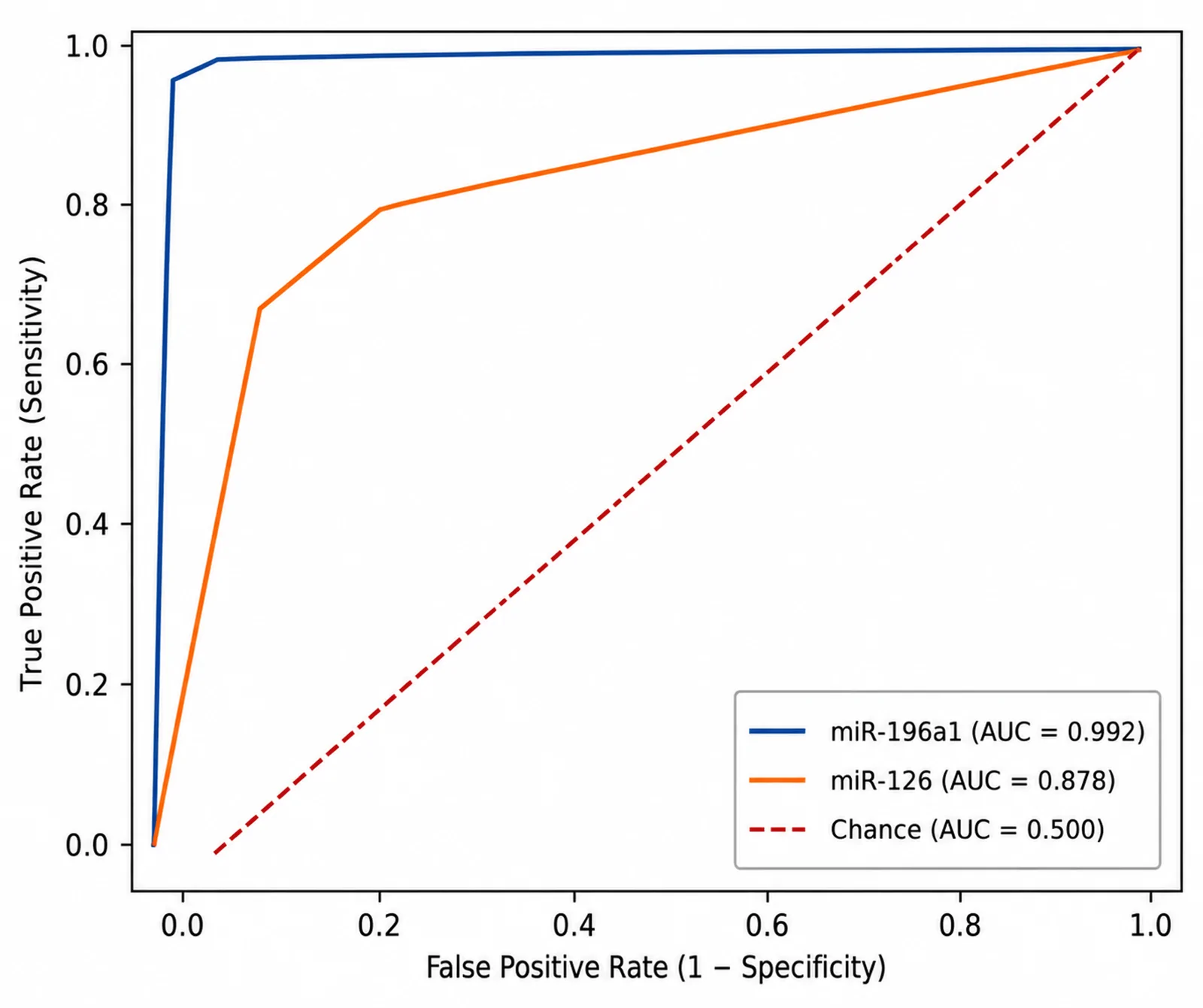
ROC Analysis of Circulating miR-196a1 and miR-126 for Distinguishing OSCC From OPMDs. Receiver operating characteristic (ROC) curves demonstrating the individual diagnostic performance of circulating miR-196a1 and miR-126 in differentiating oral squamous cell carcinoma (OSCC) from oral potentially malignant disorders (OPMDs).

ROC analysis showed an area under the curve (AUC) of 0.999 (95% CI: 0.997-1.000). Sensitivity and specificity were both greater than 98% (Figure 4).

Serum expression of miR-196a1 and miR-126

Group-Wise Analysis

**Figure 5:**
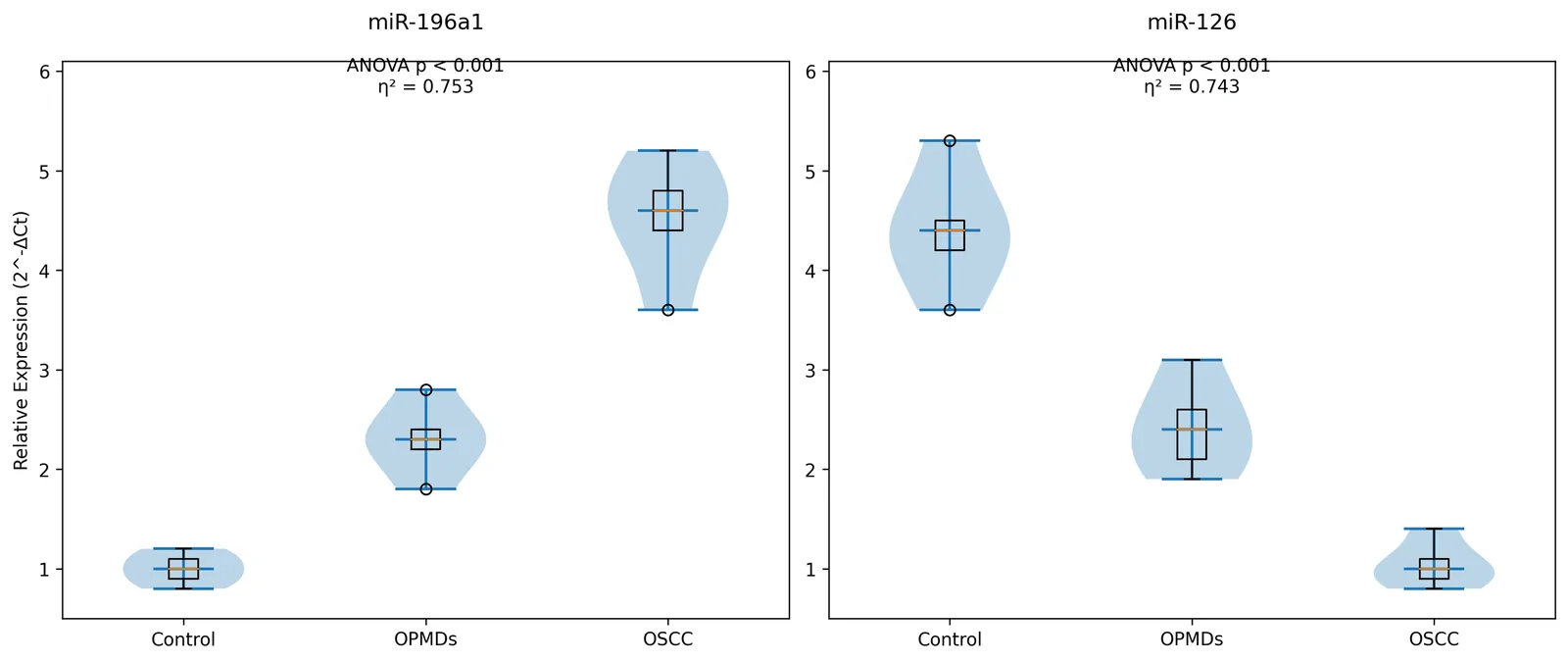
Serum Expression Profiles of miR-196a1 and miR-126 Across Study Groups Violin and box plots depict the serum expression levels of miR-196a1 and miR-126 across control, oral potentially malignant disorder (OPMD), and oral squamous cell carcinoma (OSCC) groups. The relative expression levels were determined using the 2^-ΔCt method. Box plots show the median and interquartile range, whereas violin plots represent the density of the distribution of expression values. One-way ANOVA revealed significant differences among the groups for both miRNAs (p < 0.001).

miR-196a1 expression was higher in OSCC compared to OPMDs and miR-126 expression was lower in OSCC compared to OPMDs. The differences were statistically significant (p < 0.001). One-way ANOVA showed significant differences across groups (Figure 5).

Correlation With Clinicopathological Parameters

**Figure 6:**
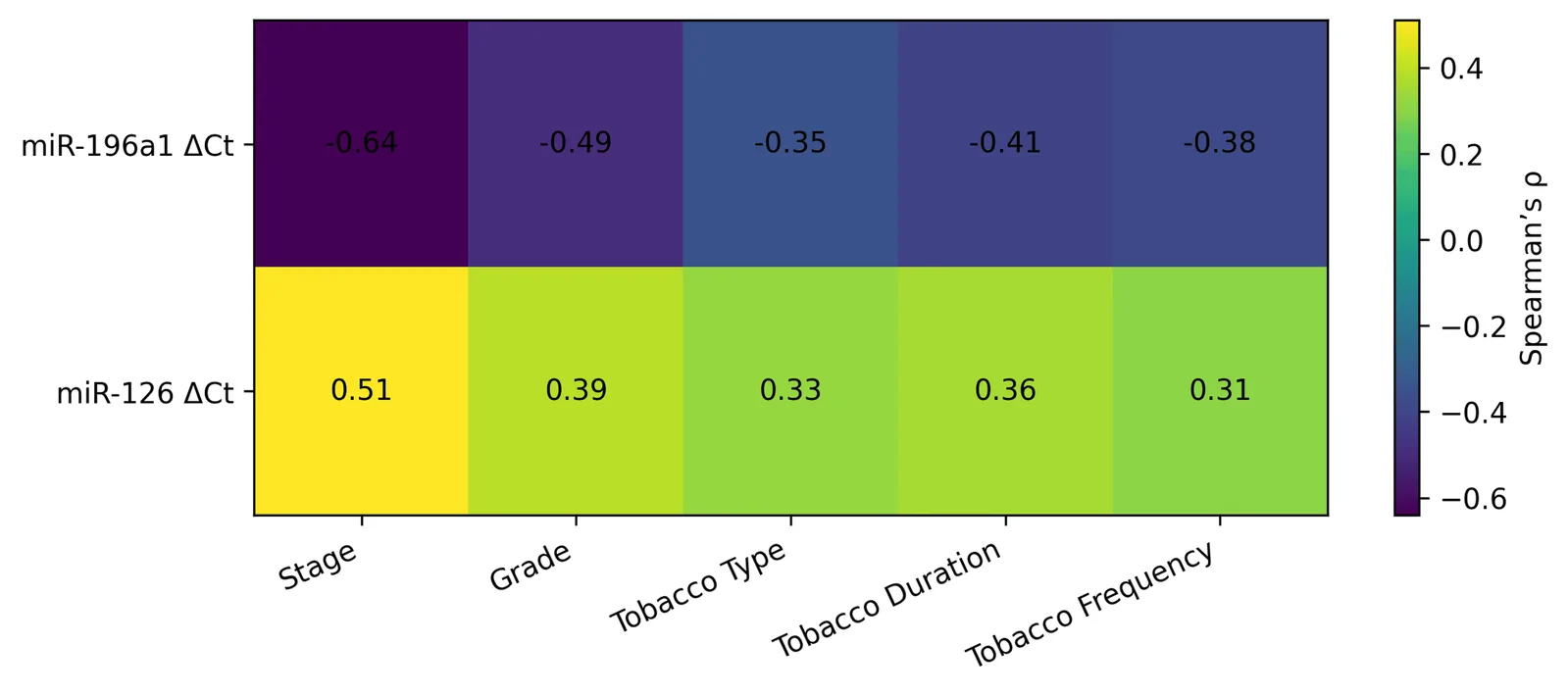
Association Between Levels of Circulating microRNA Expression and Clinical Pathological Features A heatmap illustrating the Spearman correlation coefficients (ρ) between ΔCt values of circulating miR-196a1 and miR-126 and various clinicopathological parameters, such as disease stage, histological grade, type of tobacco habit, duration of tobacco exposure, and frequency of tobacco use. Higher coefficient values indicate positive correlations, while negative coefficient values denote inverse relationships.

Spearman’s correlation analysis revealed an inverse relationship between miR-196a1 ΔCt values and disease stage (ρ = −0.642, p < 0.001), as well as with histological grade (ρ = −0.487, p = 0.006). Conversely, miR-126 ΔCt values demonstrated a positive correlation with disease stage (ρ = 0.514, p = 0.002) and histological grade (ρ = 0.392, p = 0.014). Both miRNAs were notably linked to the type of tobacco use, duration, and frequency (p < 0.05) (Figure 6).

Integrated Diagnostic Performance of miR-196a1 and miR-126

**Figure 7:**
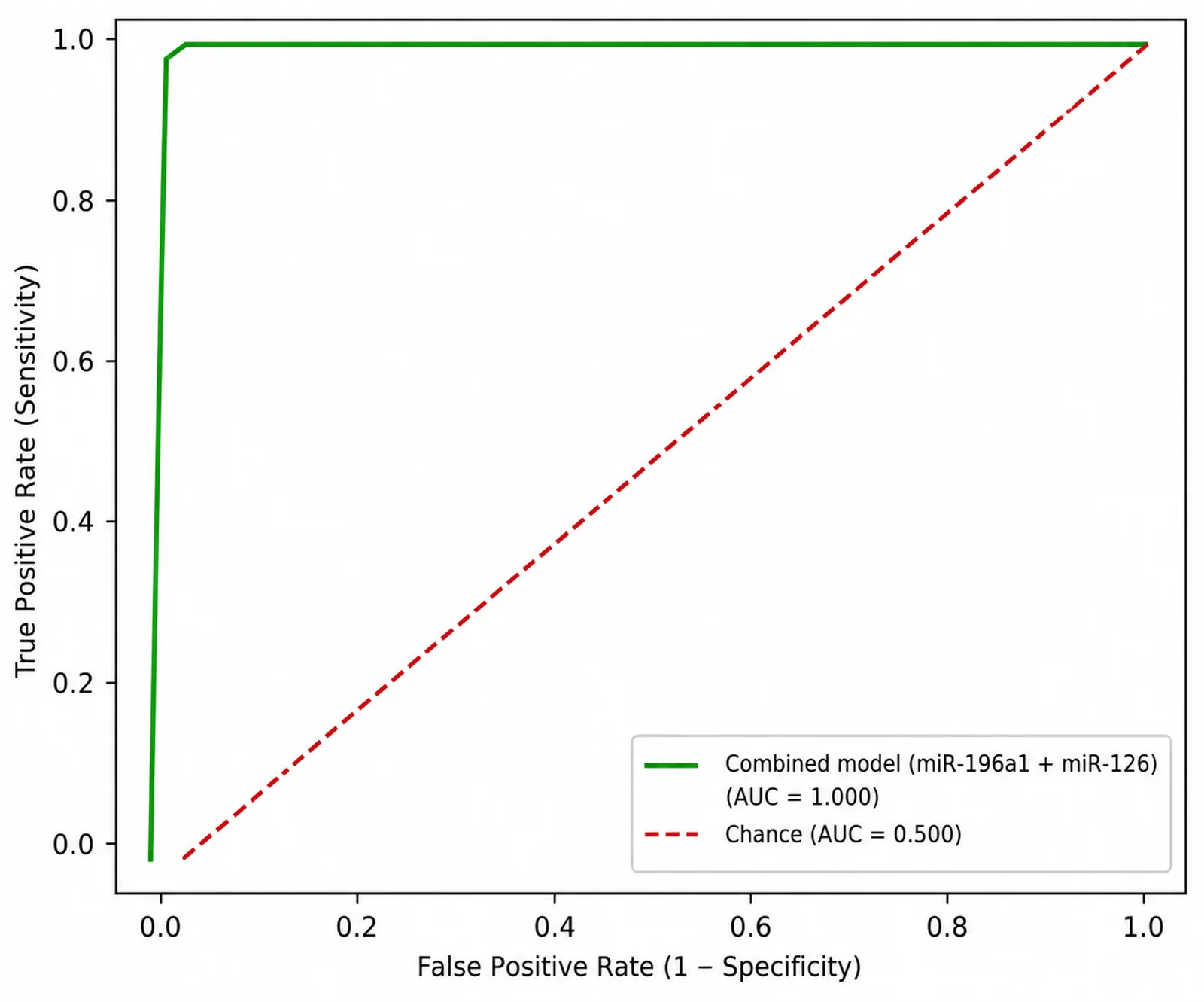
ROC analysis of the combined circulating miR-196a1 and miR-126 biomarker model for distinguishing OSCC from OPMDs. The receiver operating characteristic (ROC) curve illustrates the diagnostic effectiveness of the integrated circulating miR-196a1 and miR-126 model in distinguishing oral squamous cell carcinoma (OSCC) from oral potentially malignant disorders (OPMDs). This combined biomarker model exhibited outstanding discriminatory capability, achieving an area under the curve (AUC) of 1.000.

ROC analysis indicated a high level of diagnostic accuracy for miR-196a1, reflected by an AUC of 0.992. The integrated logistic regression model that included both miR-196a1 and miR-126 achieved an AUC of 1.000 along with a Brier score of 0.0166. The multivariable analysis revealed that both miRNAs independently contributed to the diagnostic model (Figure 7).

Concordance Between Tissue pAKT Expression and Circulating miRNA Profiles

**Figure 8:**
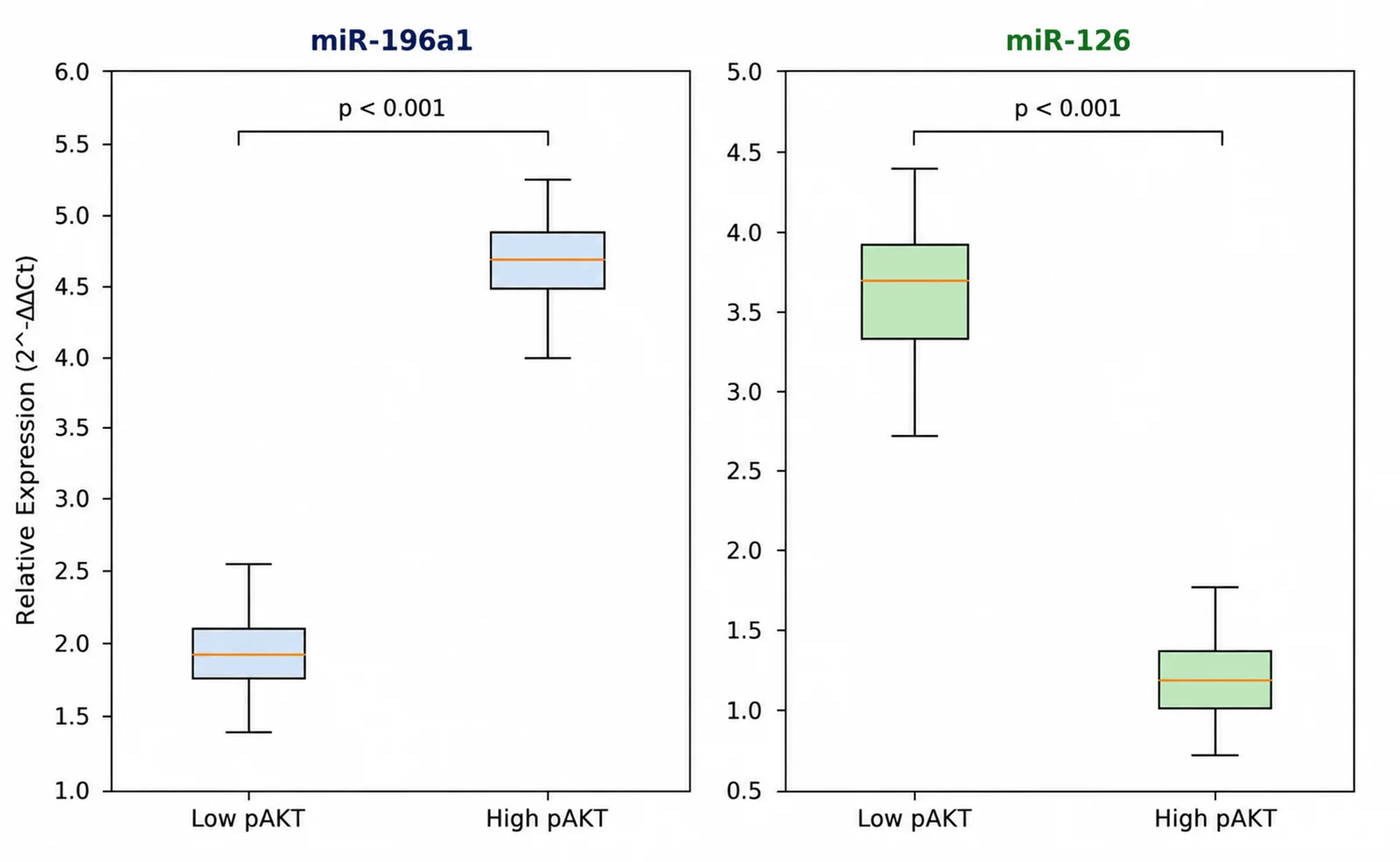
Concordance Between Tissue pAKT Expression and Circulating miRNA Profiles Box plots illustrating the expression levels of circulating miR-196a1 and miR-126 based on tissue pAKT expression status were analyzed. Samples exhibiting high pAKT expression revealed elevated miR-196a1 levels and diminished miR-126 levels in comparison to samples with low pAKT expression. The boxes indicate the interquartile range, the central lines represent the median, and the whiskers display the range of values.

OSCC cases with high pAKT expression showed increased serum miR-196a1 levels and reduced miR-126 expression. An association between tissue pAKT expression and circulating miRNA expression patterns was observed across study groups (Figure 8).

## Discussion

The present study demonstrates a biologically integrated signaling framework linking tissue-level PI3K/AKT activation with circulating miRNA dysregulation across the continuum of oral carcinogenesis. By combining immunohistochemical evidence of pAKT overactivation with reciprocal serum expression patterns of miR-196a1 and miR-126, this work strengthens the emerging concept that OSCC progression is driven by coordinated epigenetic and signaling alterations rather than isolated molecular events.

PI3K/AKT activation and malignant progression

A central finding of this study is the progressive increase in pAKT expression from premalignant lesions to invasive carcinoma, indicating stepwise activation of the PI3K/AKT signaling pathway during oral carcinogenesis. Recent molecular investigations identify PI3K/AKT as one of the most frequently dysregulated oncogenic pathways in OSCC, contributing to cellular proliferation, resistance to apoptosis, metabolic adaptation, and invasive behavior [1,2]. The strong pAKT expression observed in OSCC cases in the present study is consistent with these reports and supports the concept that sustained AKT phosphorylation represents a key molecular event driving malignant transformation rather than a secondary consequence of tumor development. Compared with recent immunohistochemical studies evaluating single biomarkers such as CD44, PD-L1, or epithelial-mesenchymal transition markers, which primarily demonstrate group-wise differences, the present findings reveal a clear signaling gradient across the disease spectrum, accompanied by high odds and risk estimates for malignant transformation. This suggests that pAKT activation may serve as a robust mechanistic discriminator between premalignant and malignant states, reinforcing its biological and diagnostic significance.

miRNA dysregulation as an upstream regulatory mechanism

In addition to tissue signaling alterations, this study demonstrates significant reciprocal dysregulation of circulating miRNAs, characterized by progressive upregulation of oncogenic miR-196a1 and suppression of tumor-suppressive miR-126. Recent literature highlights the role of miRNAs as critical epigenetic regulators of oncogenic signaling in OSCC, influencing proliferation, invasion, and tumor progression [3]. The observed increase in miR-196a1 aligns with previous experimental evidence demonstrating its oncogenic role in promoting cellular growth and malignant behavior [6]. Conversely, reduced expression of miR-126 supports its established tumor-suppressive function and its involvement in regulating PI3K pathway components, where loss of expression may enhance signaling activation and facilitate tumor progression [4,7]. The progressive reciprocal pattern observed in the present study suggests that malignant transformation involves simultaneous oncogenic gain and tumor-suppressive loss, producing a molecular environment favorable for sustained AKT pathway activation. These findings support the concept that miRNA alterations do not merely accompany disease but actively contribute to signaling dysregulation during oral carcinogenesis.

Concordance between tissue signaling and circulating biomarkers

A key novelty of this work is the demonstration of concordance between tissue pAKT activation and circulating miRNA expression. OSCC cases exhibiting strong pAKT positivity consistently showed elevated miR-196a1 and reduced miR-126 levels, indicating a biologically coherent PI3K/AKT-miRNA regulatory axis. Recent liquid biopsy studies have highlighted the diagnostic promise of circulating miRNAs but frequently emphasize the limitation of lacking a mechanistic linkage to tumor tissue [8]. By integrating tissue-based immunohistochemical data with serum biomarker profiles, the present study addresses this gap and provides stronger biological plausibility for circulating miRNAs as surrogate indicators of pathway activation. This tissue-liquid biopsy concordance significantly enhances translational relevance, suggesting that non-invasive serum biomarkers may accurately reflect intracellular oncogenic signaling events occurring within tumor tissue [9].

Diagnostic performance in the context of recent literature

The findings from this study indicate a noteworthy advancement in diagnostic performance, surpassing numerous recently reported biomarker models for OSCC. While circulating miRNA panels have shown moderate to high diagnostic accuracy in recent evaluations, issues such as variability and insufficient mechanistic integration remain prevalent [10,11]. Evaluation of the study cohort revealed that pAKT reliably differentiated malignant lesions from oral potentially malignant disorders, whereas the combined circulating miRNA model demonstrated strong discriminatory performance within the study cohort, supporting its potential utility as a complementary diagnostic tool. This enhanced accuracy likely reflects a hypothesis-driven, biologically grounded approach to biomarker selection, emphasizing functional relevance to oncogenic signaling pathways over purely statistical or data-mining strategies [12]. By integrating pathways related to tumor biology, such as pathway activation through pAKT, the gain of oncogenic miRNA (miR-196a1), and the loss of tumor suppressor miRNA (miR-126), the proposed model reflects a comprehensive molecular framework, thus improving diagnostic precision and practical application in clinical settings. Importantly, while the study achieved high diagnostic accuracy, including nearly perfect AUC values for pAKT and the combined miRNA model, these results should be contextualized within the specific study design, rather than viewed as definitive indicators of clinical performance [13,14]. The biomarkers selected were supported by robust biological evidence and illustrate various aspects of oral carcinogenesis, including activation of intracellular pathways, acquisition of oncogenic miRNAs, and depletion of tumor suppressor miRNAs. This biologically driven framework likely facilitated superior class separation among distinctly defined histopathological groups, contributing to the elevated discriminatory capability observed. The development of the model was rooted in a hypothesis-driven methodology, minimizing the risk of inflated performance due to multiple testing issues. Although internal validation processes were implemented to assess model stability, it is acknowledged that performance estimates derived from a single cohort may be overly optimistic [1,15]. While the proposed biomarker model demonstrated promising performance within the current cohort, its broader clinical utility remains to be established. Independent multicenter validation studies are warranted to confirm reproducibility, generalizability, and suitability for clinical application.

Association with tobacco exposure and biological plausibility

The significant association between biomarker dysregulation and tobacco exposure further supports biological plausibility. Tobacco-derived carcinogens are known to induce oxidative stress, genomic instability, and activation of growth-factor signaling pathways, including PI3K/AKT, contributing to malignant transformation [1]. The observed correlation between cumulative tobacco exposure and progressive biomarker alterations suggests that environmental carcinogen exposure may drive epigenetic changes that culminate in sustained signaling activation and tumor evolution. This finding enhances the clinical relevance of the proposed biomarker axis, particularly in high-risk populations with substantial tobacco burden [16].

Proposed mechanistic model

Based on the present findings and supporting literature, a unified mechanistic model can be proposed in which oncogenic upregulation of miR-196a1 and loss of tumor-suppressive miR-126 converge to enhance PI3K/AKT pathway activity, resulting in increased pAKT expression and progression from OPMDs to invasive OSCC. This integrated framework bridges intracellular signaling alterations with circulating biomarkers and provides a biologically grounded explanation for malignant progression [2,17].

Study strengths, limitations, and future perspectives

A key strength of the present investigation lies in its comprehensive assessment of the entire continuum from OPMDs to OSCC, together with the integration of tissue-based and liquid biopsy biomarkers supported by rigorous statistical analyses. Unlike many prior investigations that examine signaling pathways or circulating biomarkers independently, the present study demonstrates mechanistic continuity between tissue-level molecular events and non-invasive serum markers. However, certain limitations must be acknowledged. The cross-sectional design limits assessment of temporal causality, and external validation in larger multicentric cohorts is required to confirm reproducibility [18]. Future longitudinal studies should evaluate whether serial monitoring of circulating miRNAs can predict malignant transformation before histopathological progression becomes evident. Experimental studies investigating direct miRNA-mediated modulation of AKT phosphorylation would further strengthen mechanistic interpretation.

In conclusion, this study demonstrates that integration of tissue pAKT activation with circulating miR-196a1 and miR-126 expression provides a biologically coherent and clinically translatable framework for OSCC risk stratification. The proposed pAKT-miRNA axis offers a potential strategy for early detection, improved surveillance of OPMDs, and advancement toward precision oncology approaches aimed at identifying high-risk patients before invasive disease development.

## Conclusions

This study demonstrates an association between tissue pAKT activation and circulating miR-196a1 and miR-126 expression across the OPMD and OSCC groups studied. Integration of tissue pAKT expression with circulating miRNA profiles provides a biologically coherent and potentially useful biomarker framework for further investigation in OSCC. The findings support the potential utility of this integrated approach for distinguishing malignant from premalignant disease; however, the results should be considered exploratory, and validation in larger, prospective, and multicentric cohorts is required before clinical diagnostic or risk-stratification utility can be established.
